# Isolation and identification of *Brucella melitensis* using bacteriological and molecular tools from aborted goats in the Afar region of north-eastern Ethiopia

**DOI:** 10.1186/s12866-019-1474-y

**Published:** 2019-05-24

**Authors:** Muluken Tekle, Mengistu Legesse, Bedaso Mammo Edao, Gobena Ameni, Gezahegne Mamo

**Affiliations:** 1 Department of Veterinary Microbiology, Immunology and Public Health , College of Veterinary Medicine and Agriculture, Addis Ababa University, P. O. Box 34, Bishoftu, Ethiopia; 2 Aklilu Lemma Institute of Pathobiology, Addis Ababa University, P. O. Box 1176, Addis Ababa, Ethiopia

**Keywords:** Abortion, *B. melitensis*, Goats, Isolation, Molecular detection, Afar region, Ethiopia

## Abstract

**Background:**

Infection with *Brucella melitensis* (*B. melitensis)* is one of the most important causes of abortion in goats and sheep, and also causes severe systemic disease in exposed humans. In Ethiopia, based on seroepidemiological studies, brucellosis is known to be endemic. However, there is little information on the isolation and molecular detection of *Brucella* species in small ruminants. Therefore, the present study was conducted in the Amibara district of Afar Region of Ethiopia to isolate and molecularly detect Brucella infection in small ruminants.

**Results:**

Out of the total 64 samples cultured, eight samples (five vaginal swabs and three milk) were positive for *Brucella* species based on colony morphology, growth characteristics, modified acid fast staining and biochemical tests results. Further identification using Brucella- ladder PCR method showed that four of the isolates (three from vaginal swabs and one from milk) from goats amplified fragments of 1071 bp, 794 bp, 587 bp, 450 bp and 152 bp in band size. The molecular result combined with the microbiological and biochemical characteristics of the isolates indicated that the isolates were strains of *B. melitensis*.

**Conclusion:**

The finding of this study could suggest economic and zoonotic significance of *B. melitensis* and warrants for the need for control strategies in livestock and creation of awareness in the pastoral communities on the safe consumption of foods of animal origin and avoidance of physical contact with aborted materials.

**Electronic supplementary material:**

The online version of this article (10.1186/s12866-019-1474-y) contains supplementary material, which is available to authorized users.

## Background

Brucellosis is one of the most common bacterial diseases affecting domestic animals, humans and wildlife. It is caused by the slow-growing, gram negative, small coccobacilli bacteria of the genus *Brucella* capable of surviving and multiplying within epithelial cells, placental trophoblasts, dendritic cells and macrophages [1]. Brucellosis in livestock is mainly caused by *B. abortus*, *B. melitensis*, *B. suis*, *B. canis* and *B. ovis.* Among these species, *B. melitensis* and *B. ovis* are the common cause of brucellosis in sheep and goats [2, 3].

Brucellosis causes enormous economic losses as it affects the health of all livestock and also diminished their products [3–5]. It also poses a barrier to trade of animals and animal products, an impediment to free animal movement [6]. Currently, brucellosis has been considered as the commonest re-emerging zoonotic disease worldwide and causes a considerable human morbidity in endemic areas [5, 7–9].

Ethiopia is one of the developing countries possessing 27.35 million sheep and 28.16 million goats which are predominantly reared in the low land pastoral regions of the country [10]. However, the economic benefit obtained from these animals is by far below the expected amount. Of the different factors that limit small ruminant production, reproductive diseases such as brucellosis are the major ones particularly in the pastoral areas in Afar, Oromia and Somali regions of Ethiopia [11]. Seroprevalence studies on small ruminant brucellosis in Ethiopia showed that the prevalence ranges from 3.6 to 22.8% in pastoral areas of Afar, Oromia and Somalia Regional states [12–14]. However, all these serological studies carried so far in Ethiopia were not supplemented with isolation and identification of the *Brucella species*, which is critical for the success of control of the disease [15]. Hence, the present study was conducted to isolate and identify Brucella organisms from small ruminants in the pastoral areas of the Afar Region.

## Methods

### Study area

This study was conducted in the pastoral area of the Amibara District of the Afar National Regional State of Ethiopia. Amibara District is located in the Middle Awash Valley about 260 km to the northeast of Addis Ababa. Detailed description of the study area and the population has been given elsewhere [16]. There are about 103,959 cattle, 122,526 goats, 48,043 sheep, 3888 donkeys and 39,995 camels’ populations in the Amibara district [17]. The production system of the Afar Region is dominated by pastoralism (90%) from which agro-pastoralism (10%) is now emerging following some permanent and temporary rivers on which small scale irrigation is developed [10].

### Study design and study animals

A cross-sectional study was conducted in the Amibara district from October 2015 to April 2016. The pastoral study subdistricts or ‘*kebeles’* were selected purposively based on the population of small ruminants and accessibility for transport of specimen for bacteriological culture. Goat and sheep that had history of recent abortion (abortion occurred in last 30 days at the time of sampling), animals with retained placenta or uterine discharges were included in the study. Based on this clinical history, a total of 64 biological samples (28 milk, 27 vaginal swab, two abomasal contents and seven fetal membrane) from 60 goats and four sheep were collected for bacteriological culture.

### Study procedure and data collection

A house-to-house survey for recently aborted sheep and goats or those with history of reproductive problem were identified in each selected subdistricts or ‘*kebeles’* and the owners were interviewed using structured questionnaire (Additional file 1) regarding the duration of abortion, age of the animal, history of previous abortion and other related information. After the aim of the survey had been explained and permission obtained from the owners; milk (from those animals which provide milk during the sample collection), vaginal swab, retained fetal membrane and fetal abomasal contents from aborted foetus (if any) were collected aseptically. Vaginal swab sample were collected with sterile applicator stick into a tube containing Ames Transport Medium (HiMedia, Mumbai, India). Similarly, 10–20 ml mid-stream milk samples were collected into sterile 50 ml screw capped tube and also tissue samples were collected with sterile 50 ml screw capped tube containing sterile saline solution. All samples were kept at -20 °C at Melka Were Agriculture Research Centre (Afar Pastoral Region) until transported to the Veterinary Microbiology laboratory, College of Veterinary Medicine and Agriculture, Addis Ababa University (Bishoftu, Ethiopia) and processed for bacteriological culture.

### Sample processing and isolation

All bacteriological samples were processed under Biosafety level two (BSL2) with high personal protections as previously described [18]. Briefly, the milk samples were centrifuged at 6000 rpm for 15 min to concentrate the organism under conditions that reduce the risk of aerosol contamination to personnel, and the cream and deposit were spread on Brucella Selective Agar (HiMedia, Mumbai, India) with antibiotic supplement (FD005) as previously described [19–21]. Similarly, tissue samples were processed aseptically by removing extraneous material and chopped into small pieces, and macerated using a ‘stomacher’ or tissue grinder with a small amount of sterile phosphate buffered saline (PBS). Then, the samples were inoculated onto *Brucella* Selective Agar with antibiotic supplement (FD005) and incubated at 37 °C both in the absence and presence of 5–10% CO_2_ and cultured plates were examined for*Brucella* spp. growth on day 4 and daily for 2 weeks. *Brucella-*suspected colonies characterized by typical round, glistening, pinpoint and honey drop-like appearance [1, 19].

Finally, the presumptive isolates were checked further by Modified Ziehl-Neelsen (MZN) staining, CO_2_ requirement and biochemical tests including catalase, oxidase, urea hydrolysis, nitrate reduction, H_2_S production and growth on thionin and basic fuchsin dyes incorporated into trypticase soy agar at different concentrations as previously described [22, 23]. The isolates were collected and kept at -20 °C until processed for Bruce-ladder multiplex PCR detection.

### DNA extraction and molecular detection

Genomic DNA was extracted from all isolates through heat-lysis method of bacterial cultures, as described previously [24]. Briefly, the isolates were inoculated onto freshly prepared Trypticase -soy Broth (TSB) for 48–72 h at 37 °C. The overnight cultured *Brucella* isolates were transferred into 1.5 ml eppendorf tube. To get rid of the salts from the culture media, the suspensions were first centrifuge at max speed (14,000 rpm for 5 min) and the supernatant was discarded. Then, 250 μl of water was added in to the cell pellet and vortexed well to re-suspend the cells in the water and the re-suspended cells were lysed at 95 °C for 15 min. Then, centrifuged at 14,000 rpm for 5 min and the DNA was collected using micropipetter from the supernatant for Bruce-ladder multiplex PCR analysis. The final concentration of the extracted DNA was not determined during the procedure and it was taken as one limitation of the study.

The Bruce-ladder multiplex PCR was carried out using species-specific eight pairs of oligonucleotides PCR primers as previously described [25]. Briefly, the Bruce-ladder multiplex PCR was performed through preparing a final volume of 25 μl PCR reaction mix containing 2.5 ul of 10x PCR buffer, dNTPs (2 mM) 400 μM each one 5.0 μl, Mg2+ (50 mM) 3.0 mM 1.5 μl, *Bruce*-ladder eight pair primer cocktail (12.5 μM) 6.25 pmol each one 7.6 μl, DNA polymerase 1.5 U 0.3 μl, H_2_O (PCR-grade) 7.1 μl and 1 μl genomic DNA from the sample. The mix was vortexed briefly to ensure homogeneity of reagents and to avoid bubbles. Then, pipetted into 25 pre-labelled 0.5 ml thin walled cryovial tubes and PCR reaction mixtures were placed in a thermo cycler for amplification of the DNA through setting initial denaturation at 95 °C for 7 min, followed by 25 cycles of template denaturation at 95 °C for 35 s, primer annealing at 64 °C for 45 s and primer extension at 72 °C for 3 min, followed by a final extension at 72 °C for 6 min.

After PCR amplification, 2 μl of PCR product and 8 μl of bromophenol blue (loading buffer) were loaded into wells in 1.5% agarose gel in Tris-HCl, boric acid and Ethylene diethyl tetracetic acid (EDTA) (TBE) buffer in a cuvette flooded with TBE slightly covering the gel. One hundred base pairs DNA ladder /1 kb plus DNA ladder was used as molecular marker. Sterile ultrapure water was used as negative control and *B. suis* 1330 bv1, *B. ovis* 63/290*, B. melitensis* Rev.1 (vaccine strain) and *B. abortus* RB51 (APHA, UK) were used as positive controls. To visualize bands, the gel was stained with ethidium bromide DNA gel stain. The electrophoresis equipment was set to run at 130 V for 50 min after the electrodes applied accordingly [25]. Finally, the gel was visualised under UV light and the bands were observed and recorded.

### Serological tests

Sera collected from aborted goat cases with confirmed bacteriological and molecular positive results were tested for sero-positivity using modified Rose Bengal test and Complement fixation test according to OIE procedures [18] and sera were also tested using competitive ELISA as per the manufacturers’ instruction (Svanova, Brucella-ab c-ELISA Uppsala Business Park. Uppsala, Sweden).

## Results

### Colony characteristics and isolation of Brucella organisms

Growth of colonies were first observed on *Brucella* selective agar as early as 72 h and majority of the isolates were obtained after 96 h of incubation at 37 °C without CO_2,_ whereas no growth was observed under CO_2_ supply. When examined under stereomicroscope, the colonies showed characteristic honey-like appearance with very small, glistening, smooth, round and pin-point morphology. The cellular characteristics of the isolates showed gram negative small coccobacilli arranged singly and in pairs during MZN stain.

Based on biochemical reactions like; urea hydrolysis test and growth on basic fuchsin dye eight (12.5%) *Brucella* isolates were recovered from the 64 samples. Of these, three isolates were from milk samples while five were from vaginal swabs collected from goats. However, no of isolate was recovered from aborted sheep, fetal abomasal contents and fetal membranes (Table 1).

**Table 1:** Types of samples and *Brucella* isolates

Sample Type	Animal Species		Sample cultured	Isolates	Percentages (%)
	Sheep	Goat			
Milk	2	26	28	3	10.71
Vaginal swab	–	27	27	5	18.51
Fetal abomasal content	–	2	2	0	0
Fetal membrane	2	5	7	0	0
Total	4	60	64	8	12.5

### Identification of Brucella organism using Bruce-multiplex PCR

**Fig. 1:**
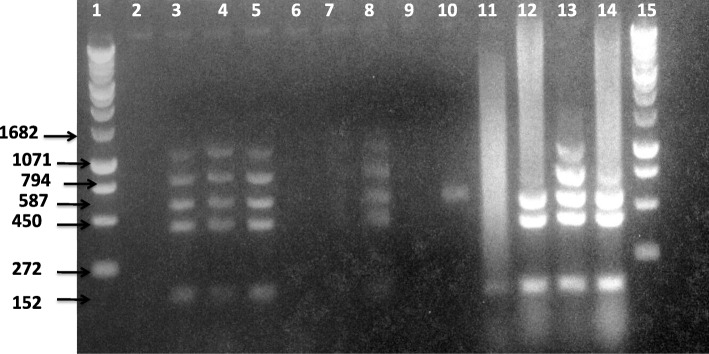
Result of multiplex PCR conducted on *Brucella* isolates. Lanes 1 and 15, 1 Kb plus DNA ladder (Invitrogen); lane 2, negative control (H_2_O); lanes 3–5 and lane 8, isolates of *B. melitensis*; Lane 6, 7 9 and 10 negative samples; Lane 11–14, reference positive control (DNA from *B. suis* 1330 bv1, *B. ovis* 63/290*, B. melitensis* Rev.1 (vaccine strain) and *B. abortus* RB51)

From the eight isolates, the Bruce-ladder multiplex PCR assay confirmed four isolates of *B. melitensis*; of which three from vaginal swab and one from milk samples. All yielded amplicons of 1071 bp, 794 bp, 587 bp, 450 bp and 152 bp bands but did not produce the 1682 bp band size fragment and it corresponded to the bands amplified by the reference *B. melitensis* strain used as control. Serological examination of all the four goats from which *B. melitensis* was isolated were seropositive with Rose-Bengal Plate test, Complement Fixation Test and Enzyme Linked Immuno Sorbent Assay (data not shown). On the other hand, the remaining four samples were negative for any amplified amplicons (Fig. 1). In the positive control, *B. abortus* RB51 was distinguished by the presence of amplicons of 794 bp, 587 bp, 450 bp and 152 bp. On the other hand, *B. ovis* 63/290 was distinguished by the presence of amplicons of 587 bp, 450 bp and 152 bp. Both *B. abortus* RB51 and *B. suis* 1330 bv1 controls were also characterized by absence of 1071 bp and 1682 bp amplicons. In this experiment, *B. suis*1330 bv1 control showed smearing of band except presence of amplicon of 152 bp which might be caused by degradation of DNA.

## Discussion

Seroepidemiological studies on brucellosis in various parts of Ethiopia indicated that the disease is widespread among small ruminant populations [11–14]. However, little has been done on bacteriological isolation of the *Brucella* species from clinically aborted goat cases using microbiological culture method which is a gold standard confirmatory diagnostic method [18]. Moreover, the identification of the *Brucella* species and biovariant circulating in the livestock is a prerequisite for designing relevant vaccine for control and eradication of brucellosis in a country. To our knowledge except a single report [15], hitherto there is no reliable information on isolation and molecular detection of *Brucella* species from clinical samples. In the present study, we isolated *B. melitensis* from vaginal swab and milk samples of recently aborted goats using bacteriological culture method.

Further analysis of the bacteriologically confirmed eight *Brucella* species using multiplex Bruce-ladder PCR suggested that the four isolates were *B. melitensis*; while the remaining bacteriologically positive four *Brucella* species test negative for *Brucella* by PCR, this might be due to the drawback of the DNA extraction method used, i.e. using heat lysis method of bacterial cell culture as indicated in similar studies, such method can result in very low concentration of the DNA which consequently result in false negative in PCR as compared to commercial kit based DNA extraction method [26]. Although the PCR using the DNA from the four isolates amplified the characteristics four fragments of 1071 bp, 794 bp, 587 bp, 450 bp and 152 bp band size, the commonly amplified 1682 bp fragment in most *B. melitensis* strain was absent, making them similar to the *B. ovis* as described previously [27]. However, the typical microbiological and biochemical characteristics including smooth colony morphology, being oxidase positive, urease positive and its agglutination with M-specific anti-sera strongly suggested that the isolates belong to the strains of *B. melitensis* rather than *B. ovis*. The fact that the isolate were collected from recently aborted goats with typical clinically signs of Brucellosis, we believe that the isolates circulating in the study area might be different from the previously characterized strains of *B. melitensis.* As future direction, further characterization of the isolates using advance molecular techniques including whole genome sequencing will be carried out to confirm and verify the results.

The isolation and confirmation of *B. melitensis* from goats in the present study is consistent with previous report where two *B. melitensis* were isolated from tissue samples (inguinal lymph nodes, testes, spleen and lung) collected from 14 strong seropositive goats [15], which suggests that *B. melitensis* could be the predominant cause of brucellosis in goats of Ethiopia. Similar to our report, *B. melitensis* was isolated from tissue and blood samples collected from a recently aborted goats and sheep in Jordan [28], from vaginal exudate of recently aborted goat in Mexico [29], from vaginal swabs, spleen, uterine fluid collected from seropositive goats in Peninsula Malaysia [30], and from milk collected from goats and sheep in Iran [31].

In the present study, out of the 64 samples collected from the study animals with clinical cases of reproductive problem, only eight (12.5%) samples showed growth of colonies*.* This could be due to the fastidious nature of *Brucella* species, stage of the disease and quantity of shed bacteria through milk or uterine discharge that might affect the isolation rate [32]. However, the observed prevalence of isolates from milk (37.5%) and vaginal swab (62.5%) as well as the confirmed prevalence of *B. melitensis* species from milk (33.3%) and from vaginal swab (60%) in the present study is higher than the prevalence of isolates and confirmed prevalence of *B. melitensis* species from milk and vaginal swab collected from goats in Iran [23], which might be related to the prevailing risk factors for infection in the study areas. On the other hand, the observed prevalence is comparable to the prevalence reported by Bamaiyi et al. [30]. The overall low *Brucella* culture positivity from goats and sheep with clinical cases of reproductive problem in the present study might also suggest that the existence of other causes of abortion other than brucellosis in the study animals and warrants further studies.

The isolation and confirmation of *B. melitensis* from milk and vaginal swab of goats in the present study, also strongly indicates the zoonotic importance of *B. melitensis* in pastoral communities of the study area. Since the majority of population consume unpasteurized goat milk and exposed to direct skin contact with uterine discharge, retained placenta aborted foetus when goats give birth. The most common means of transmission of brucellosis from animals to humans is through the consumption of unpasteurized milk and milk products [33]. Among other *Brucella* species, *B. melitensis* is mostly responsible for human brucellosis and several investigators have isolated this species from clinical samples obtained from patients suspected for brucellosis [34–36]. Although further studies on the status of *Brucella* infection in humans are needed, it is very important to suspect brucellosis among febrile patients, create awareness among health professionals and increase laboratory capacity for isolation and characterization of *Brucella* species in order to provide appropriate diagnosis and treatments in the present study area.

## Conclusion

The finding of the present study could suggest that *B. melitensis* is one of the *Brucella* species circulating among small ruminants and could be the major cause of abortion in goats in Amibara District of Afar Region. Hence, the results of this study warrants for the need for appropriate control strategies to reduce the economic and zoonotic impacts of Brucellosis.

## Additional file

Additional file 1: Questionnaire for small ruminant owners- small ruminant Brucellosis Project-the questionnaire to assess the duration of abortion, history of previous abortion, other related information and to assess the cultural habits of the owners on consumption of animal products and other related risk factors. In addition it also contain data collection format on those aborted animals used as source of bacteriological sample sources. (DOCX 19 kb)

## Abbreviation

AAU: Addis Ababa University
ALIPB: Aklilu Lemma Institute of Pathobiology
CVMA: College of Veterinary Medicine Agriculture
ITM: Institute of Tropical Medicine
