# Supplementary material for: Isolation and identification of Brucella melitensis using bacteriological and molecular tools from aborted goats in the Afar region of north-eastern Ethiopia
Source: BMC Microbiol. 2019 May 24;19:108. doi: 10.1186/s12866-019-1474-y (PMC6534919; doi:10.1186/s12866-019-1474-y)
Supplement: Supplementary file 1 — Questionnaire for small ruminant owners- small ruminant Brucellosis Project-the questionnaire to assess the duration of abortion, history of previous abortion, other related information and to assess the cultural habits of the owners on consumption of animal products and other related risk factors. In addition it also contain data collection format on those aborted animals used as source of bacteriological sample sources. (DOCX 19 kb) [file 12866_2019_1474_MOESM1_ESM.docx]

**Date:** October 2015

**Questionnaire format (Brucella project) for small ruminant owners in the Afar Region of northeastern Ethiopia**

**Name of the owner**______________________**Gender**_______**Age**_______**Address**________

**Education level**___________________

1. How do you keep your sheep and goats at day time?

A. Mixed with other livestock B. Separated from other livestock

2. How do you keep sheep and goats at night time?

A. Mixed with other livestock B. Separated with other livestock

3. Can your flocks contact with other flocks at grazing? A. Yes B. No

4. Who is responsible for milking sheep and goats?

A. Wife B. Husband C. Other house member

5. How do you consume sheep and goat milk?

A. Raw B. Boiled C. yoghurt D. cheese

6. Do you slaughter sheep and goats at home?

A. Yes B. No

7. If yes, for what purpose? A. Home consumption B. Ceremony purpose C. Group share D. Emergency slaughter

8. How do you consume sheep and goats meat?

A. Cooked B. Raw C. Both

9. What is your role in sheep and goats husbandry?

A. Flock feeding and watering B. Milking C. Delivery assisting D. Treating when sick

10. Do you own recently aborting sheep and goats? A. Yes B. No

11. If yes, when did the abortion occur in this aborted goat/sheep? ______________week/months ago

*In cases of abortion occurred more than one sheep/goat additional it will be recorded in additional format (page 4)

12. What is the age of the goat/sheep when abortion occurred? year

13. At what stages of pregnancy does the abortion occurred? A. In the first two months B. In the mid two months of pregnancy C. In the last two months of pregnancy (last trimester)

14. Is there any previous abortion occurred? A. Yes B. No

15. How do you dispose aborted fetus/fetal membrane?

A. Throwing on the field B. Offering dogs to eat C. Burring/burning D. Do not bother about it

16. Do you have any male sheep or goat with scrotal or joint swollen? A. Yes B. No

17. What type of breeding method do you use? A. Natural B. Artificial insemination

C. Both

18. Do you have a sick person among your family? A. Yes B. No

15. If yes, does the sick person encountered with the following health problems lasting for 15 days?

A. Headache……….. B. Back pain……… C. Joint pain……. D. Weakness……………

E. Intermittent fever…….. F. Night sweating……… G. Pain on testis if male….

19. Does the sick person visit a hospital or a clinic? A. Yes B. No

20. Do you know disease which can be transmitted from sheep and goats to human? A. Yes B. No

21. What do you know about Brucellosis or local name “Hahayita”? ..................................................

22. If you know Brucellosis or local name “Hahayita”, how does it transmit to human? …………………………… …………….

23. Do you know other diseases that can be cause abortion on the last trimester (still birth)?

A. Yes B. No

If yes, can you mention some of them? ..............................................................

**Date…………**

**Bacteriological sample collection format in the Afar Region of northeastern Ethiopia, prepared by Afar *Brucella* Project**

| S/N | Code | Animal name | Owners name | District | Village | Spp. | Sex | Age | BCS | Parity number | Abortion history | Time of abortion  -days ago | Type of sample collected | | | | |
| --- | --- | --- | --- | --- | --- | --- | --- | --- | --- | --- | --- | --- | --- | --- | --- | --- | --- |
|  |  |  |  |  |  |  |  |  |  |  |  |  | Recently aborted fetus | Vaginal swab | RFM | Milk | Other |
|  |  |  |  |  |  |  |  |  |  |  |  |  |  |  |  |  |  |
|  |  |  |  |  |  |  |  |  |  |  |  |  |  |  |  |  |  |
|  |  |  |  |  |  |  |  |  |  |  |  |  |  |  |  |  |  |
|  |  |  |  |  |  |  |  |  |  |  |  |  |  |  |  |  |  |
|  |  |  |  |  |  |  |  |  |  |  |  |  |  |  |  |  |  |
|  |  |  |  |  |  |  |  |  |  |  |  |  |  |  |  |  |  |
|  |  |  |  |  |  |  |  |  |  |  |  |  |  |  |  |  |  |
|  |  |  |  |  |  |  |  |  |  |  |  |  |  |  |  |  |  |
|  |  |  |  |  |  |  |  |  |  |  |  |  |  |  |  |  |  |
|  |  |  |  |  |  |  |  |  |  |  |  |  |  |  |  |  |  |
|  |  |  |  |  |  |  |  |  |  |  |  |  |  |  |  |  |  |
|  |  |  |  |  |  |  |  |  |  |  |  |  |  |  |  |  |  |
|  |  |  |  |  |  |  |  |  |  |  |  |  |  |  |  |  |  |
|  |  |  |  |  |  |  |  |  |  |  |  |  |  |  |  |  |  |
|  |  |  |  |  |  |  |  |  |  |  |  |  |  |  |  |  |  |
|  |  |  |  |  |  |  |  |  |  |  |  |  |  |  |  |  |  |
|  |  |  |  |  |  |  |  |  |  |  |  |  |  |  |  |  |  |
|  |  |  |  |  |  |  |  |  |  |  |  |  |  |  |  |  |  |
|  |  |  |  |  |  |  |  |  |  |  |  |  |  |  |  |  |  |
|  |  |  |  |  |  |  |  |  |  |  |  |  |  |  |  |  |  |
|  |  |  |  |  |  |  |  |  |  |  |  |  |  |  |  |  |  |

S/N; Serial number, Spp; Species, BCS; Body condition, RFM; Retained fetal membrane
